# Tracking the onset date of the community spread of SARS-CoV-2 in western countries

**DOI:** 10.1590/0074-02760200183

**Published:** 2020-09-04

**Authors:** Edson Delatorre, Daiana Mir, Tiago Gräf, Gonzalo Bello

**Affiliations:** 1 Universidade Federal do Espírito Santo, Centro de Ciências Exatas, Naturais e da Saúde, Departamento de Biologia, Alegre, ES, Brasil; 2 Universidad de la República, Centro Universitario Regional del Litoral Norte, Unidad de Genómica y Bioinformática, Salto, Uruguay; 3 Fundação Oswaldo Cruz-Fiocruz, Instituto Gonçalo Moniz, Salvador, BA, Brasil; 4 Fundação Oswaldo Cruz-Fiocruz, Instituto Oswaldo Cruz, Laboratório de AIDS e Imunologia Molecular, Rio de Janeiro, RJ, Brasil

**Keywords:** SARS-CoV-2, community spread, Europe, America

## Abstract

Severe acute respiratory syndrome coronavirus 2 (SARS-CoV-2) rapidly spread around the world during 2020, but the precise time in which the virus began to spread locally is difficult to trace for most countries. Here, we estimate the probable onset date of the community spread of SARS-CoV-2 for heavily affected countries from Western Europe and the Americas on the basis of the cumulative number of deaths reported during the early stage of the epidemic. Our results support that SARS-CoV-2 probably started to spread locally in all western countries analysed between mid-January and mid-February 2020, thus long before community transmission was officially recognised and control measures were implemented.

A novel Betacoronavirus, designated severe acute respiratory syndrome coronavirus 2 (SARS-CoV-2), was identified as the causative agent of a severe acute respiratory disease [now known as Coronavirus disease 2019 (COVID-19)] in Wuhan, Hubei province, China, in December 2019.^1^ ^,^ ^2^ In the following weeks, SARS-CoV-2 rapidly spread around the world, infecting more than 12 million people and causing more than 500,000 deaths as of July 10th, 2020.^3^ The exponential growth of the COVID-19 pandemic has overloaded hospitals and governments’ response measures have disrupted social contacts for > 1 billion inhabitants worldwide.

Genomic analyses traced back the origin of SARS-CoV-2 in China to late November 2019,^4^ consistent with epidemiological findings that show local viral transmission in Wuhan by the middle December 2019.^5^ The first infections of SARS-CoV-2 identified in Europe and the United States of America (USA) were documented in January 2020, related to travelers returning from China and their contacts; while the firsts cases of viral community transmission in those regions were only documented between the middle and late February 2020.^6^ ^,^ ^7^ The precise onset date of the community transmission of SARS-CoV-2 in most countries, however, is difficult to estimate. The high proportion of asymptomatic/presymptomatic infectious individuals coupled with limited testing might have facilitated the undocumented dissemination of the novel coronavirus between and within countries before its detection by public health systems.^8^ ^,^ ^9^ Consistent with this hypothesis, one study retrospectively identified the presence of SARS-CoV-2 in a patient with no history of recent travel that was hospitalised in France in late December 2019.^10^ This early detection of the virus in Europe in December 2019, however, did not demonstrates that community transmission chains detected several weeks later actually originated from this very first case.

Genomic epidemiology has been used to track the geographic spread of SARS-CoV-2 and to estimate when community transmission of SARS-CoV-2 was first established in western countries. Some studies traced the onset date of the largest SARS-CoV-2 community transmission clusters to around early February in Italy and Spain, mid-February in Washington State and New York city (NYC), and from late February onwards in Belgium and the United Kingdom (UK).^11^ ^,^ ^12^ ^,^ ^13^ ^,^ ^14^ ^,^ ^15^ ^,^ ^16^ ^,^ ^17^ These results supports that rapid interventions prevented onward transmission of early symptomatic imported cases detected in Europe and the USA in January 2020 and that community outbreaks were seeded by unnoticed introductions occurred after detection of first travel-associated cases.^12^ ^,^ ^16^ ^,^ ^18^ Other genomic studies, however, support a period of undetected community spreading of SARS-CoV-2 since mid-January 2020 in Europe, late January or early February 2020 in the USA (Washington and Illinois) and early or mid-February 2020 in Brazil.^13^ ^,^ ^19^ ^,^ ^20^ ^,^ ^21^ ^,^ ^22^ ^,^ ^23^ According to these studies, community outbreaks in Europe and the Americas were seeded by unnoticed viral introductions that probably occurred before detection of first travel-associated cases and long before description of the firsts cases of community transmission.

These contrasting findings expose the limitations for the accurate estimation of the onset date of domestic transmission of SARS-CoV-2 within countries (or cities) based solely on genetic data, particularly in Europe and the Americas where many outbreaks were seeded by identical (or closely related) viral strains.^24^ The very low levels of genetic diversity of SARS-CoV-2 genomes sampled from different regions combined with the uneven geographic sampling make the evolutionary patterns inferred from early genomic data highly uncertain.^4^ ^,^ ^25^ ^,^ ^26^ Here, we aimed to develop a simple inference method to estimate the probable onset date of the community spread of SARS-CoV-2 in different countries from the time series of cumulative number of reported deaths during the early stage of the epidemic. The reported number of deaths provides a more reliable tracker of the SARS-CoV-2 epidemic’s progress within a country than counts of diagnosed cases because there are less affected by substantial underreporting.^27^ Furthermore, the cumulative number of reported deaths represents a time-delayed tracker of the SARS-CoV-2 epidemic (median time between infection and death of around three weeks) and thus provides valuable information on early epidemic dynamics even when data is obtained after implementation of control measures to reduce the viral spread.^28^ ^,^ ^29^

To infer the probable onset date of the community spread of SARS-CoV-2 in a given location, we assumed that: (a) as soon as the virus starts spreading locally, the epidemic starts to grow exponentially and the cumulative number of deaths starts to increase exponentially 20 days later; (b) the rate of exponential growth of the number of deaths remains roughly constant during the epidemic early weeks; and (c) the infection fatality ratio of SARS-CoV-2 is around 1%.^28^ ^,^ ^29^ For this study, we recovered daily death counts of COVID-19 from China and from those countries from Western Europe [Belgium, France, Germany, Italy, Netherlands, Spain, United Kingdom (UK)], North America (New York, USA) and South America (Brazil) that were most heavily affected until 5th April 2020.^30^ To capture the early period of constant exponential growth of virus transmission in each country while minimising both the impact of the low detection rate during the epidemic’s first days and of control measures implemented at later times, we set the start point of the time series to the day when the cumulative number of deaths was above four and then counted for a maximum time period of 15 days after that. Then, for each location, we used the Wald-Wolfowitz runs test statistic to select the time interval where the epidemic growth rate adjusted to the exponential function:

$$N(t)=N_{0e}{}^{kt}$$

where N(t) = number of infected individuals at time *t*, N_0_ = size of the epidemic in *t*_0_, and k = exponential growth constant. We estimate the epidemic doubling time (EDT) as EDT = ln(2)/k. Finally, to accommodate different scenarios in which the local epidemic might have been seeded by single or multiple introductions, we projected back the *t* when the number of total infected individuals was equal to one (TN1), 10 (TN10) and 100 (TN100). All statistical analyses were performed using Graph Pad v6 (Prism Software, La Jolla, California, USA).

Our analyses identified an initial period in each country, varying from nine to 15 days after the fourth death, during which the growth of deaths fits (r^2^ ≥ 0.95) an exponential curve (Fig. 1) and data were consistent with the assumption that transmission rates remain nearly unchanged in the selected period [Supplementary data (Table)]. When additional time points beyond the selected period were included, we observed a significant departure from unconstrained exponential growth and lower mean estimates of EDT were obtained (data not shown), which likely reflects the impact of control measures implemented across countries.^31^ ^,^ ^32^ ^,^ ^33^ During the early growth phase here selected, the mean EDT of SARS-CoV-2 ranged between 2.2 and 2.9 days for the whole set of analysed countries (Fig. 1), which is in line with previous estimates for China, Europe and the USA.^34^ ^,^ ^35^ ^,^ ^36^ Of note, the mean EDT of the SARS-CoV-2 estimated in Brazil was comparable to those estimated in countries from the Northern hemisphere. This supports the notion that population immunity is a much more fundamental driver of early pandemic SARS-CoV-2 dynamics than underlying demography and environmental factors.^37^ It is also possible that similar EDT estimated from death counts across countries resulted from the complex interaction of demographic and/or environmental factors with differences in the availability of health-care facilities.^29^ ^,^ ^38^ ^,^ ^39^ ^,^ ^40^

**Fig. 1:**
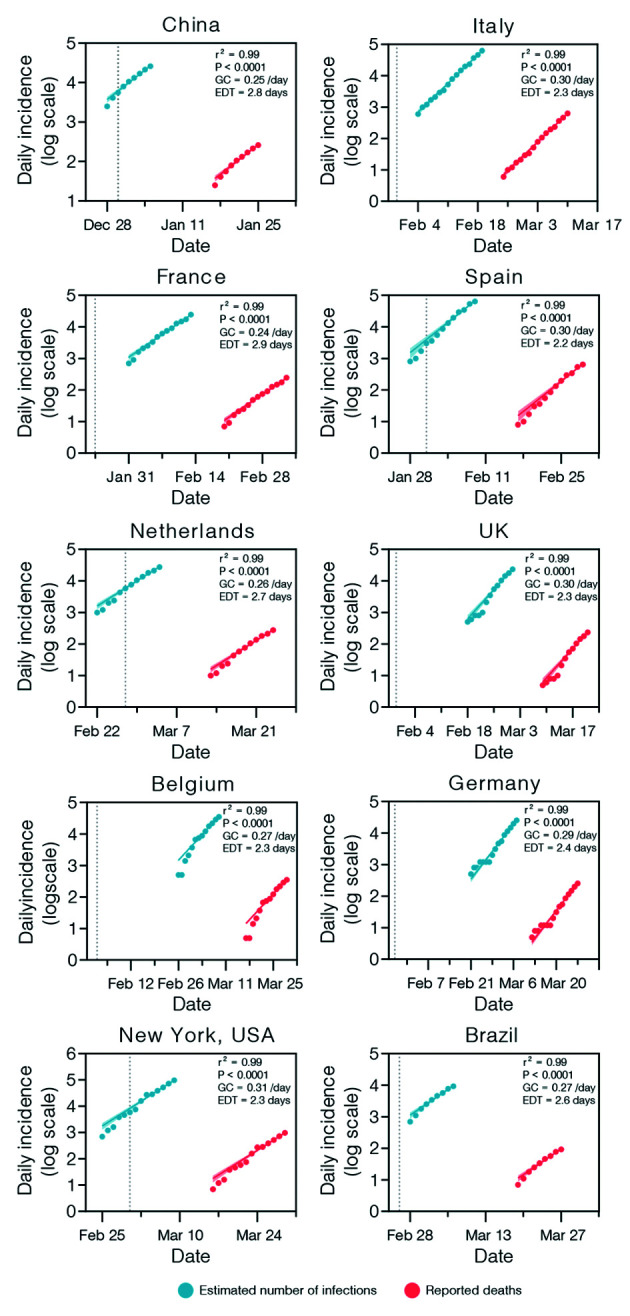
estimation of the exponential growth parameters of severe acute respiratory syndrome coronavirus 2 (SARS-CoV-2) epidemics over time in selected countries. Red and blue symbols represent the log-transformed daily counts of new deaths and the estimated number of infections, respectively, for each country. The data were fitted on an exponential growth curve (red and blue lines, with the shaded area representing the 95% confidence intervals). The goodness of fit (r²), p-value, growth constant (GC), and estimated doubling time (EDT) for each country are presented for each graph. The gray dotted line indicates the date of the 1st reported SARS-CoV-2 case in each country.

Using our approach to estimate the onset date of local transmission of SARS-CoV-2, we traced the TN1 to late November 2019 in China, to mid-January 2020 in Italy and between mid-January and early February 2020 in other western countries (Fig. 2, Table). The estimated TN1 in China is fully consistent with molecular clock calibrations obtained from early SARS-CoV-2 genomic data from Wuhan (Table).^4^ Our estimate of the onset date of community transmission of SARS-CoV-2 in Italy is also consistent with the estimated time of the most recent common ancestor (T_MRCA_) of the predominant European SARS-CoV-2 clade B.1, that most likely arose in Italy and rapid spread to other European countries (Table).^12^ ^,^ ^13^ ^,^ ^21^ The TN1 projections for other western countries, by contrast, recovered dates that were older than those estimated from genomic data (Table). These results indicate that the TN1 projection method provide quite accurate results for locations like Wuhan (China) and Northern Italy where the epidemic was mostly seeded by a single founder event; but not for most urban hubs in Europe and the Americas where the epidemic was driven by multiple independent seeding events.

**Fig. 2:**
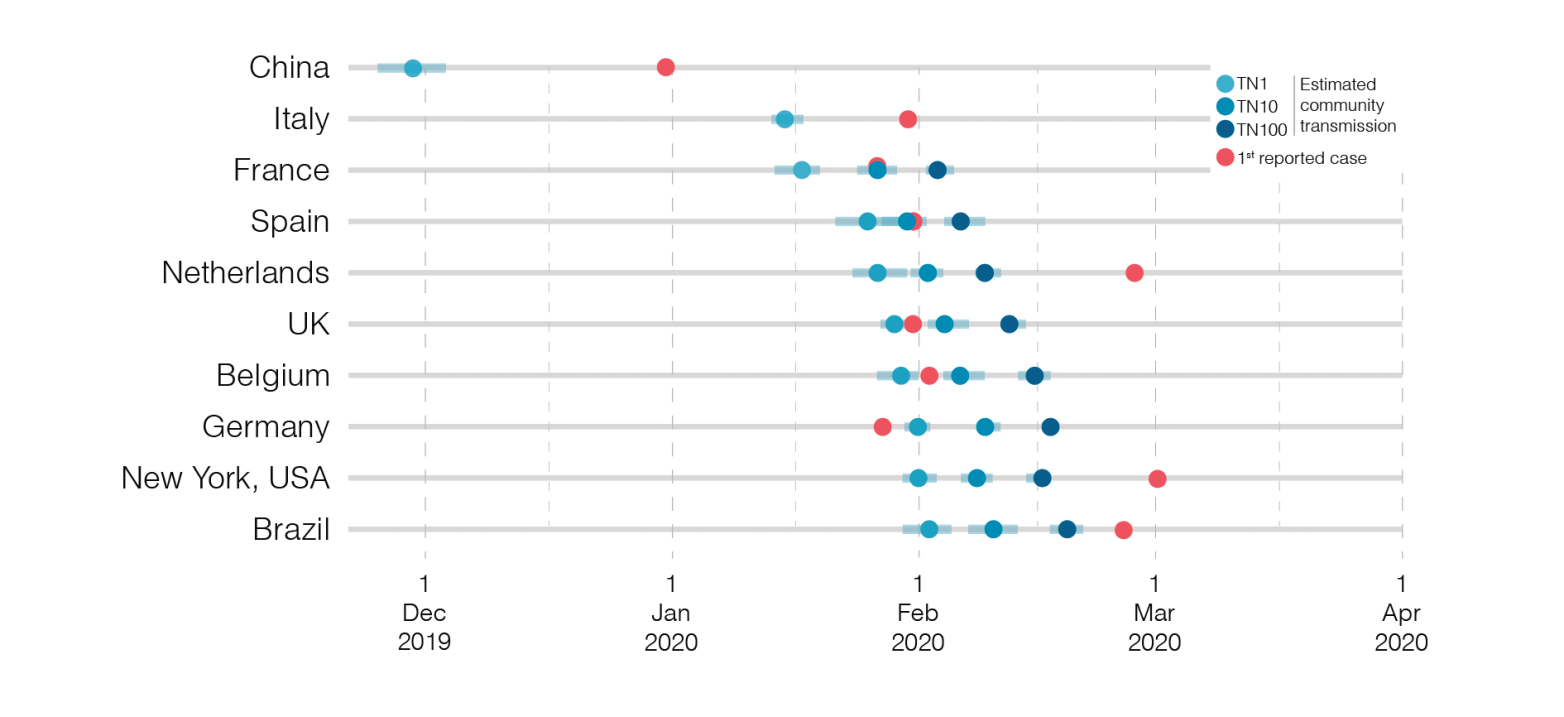
timeline of the Coronavirus disease 2019 (COVID-19) pandemic in the analysed countries. The red dot represents the date of the 1st reported severe acute respiratory syndrome coronavirus 2 (SARS-CoV-2) case in each country. Our estimates of the onset date of community transmission considering one (TN1), 10 (TN10), and 100 (TN100) seeders are represented by the blue dots according to the legend. The blue bars indicate the 95% confidence intervals of date estimates.

**TABLE:** Coronavirus disease 2019 (COVID-19) epidemiologic timeline and implementation of related control measures in selected countries

Country/City	1st reported case*	1st recorded death*	Control measures*		Estimated community transmission			
			Internal movement	International travels	Molecular clock (95% HPD)	EGM (95% CI) 1 seeder	EGM (95% CI) 10 seeders	EGM (95% CI) 100 seeders
China	31-Dec-19	09-Jan-20	23-Jan-20	28-Mar-20	01-Dec-19^1^ (15-Nov - 13-Dec)	30-Nov-19 (25-Nov - 03-Dec)	-	-
Italy	30-Jan-20	21-Feb-20	23-Feb-20	31-Jan-20	20-Jan-20^2^ (10-Jan - 29-Jan)	13-Jan-20 (12-Jan - 15-Jan)	-	-
France	24-Jan-20	15-Feb-20	12-Mar-20	17-Mar-20	-	15-Jan-20 (12-Jan - 17-Jan)	24-Jan-20 (22-Jan - 26-Jan)	03-Feb-20 (02-Feb - 04-Feb)
Spain	31-Jan-20	13-Feb-20	11-Mar-20	10-Mar-20	14-Feb-20^3^ (04-Feb - 23-Feb)	23-Jan-20 (19-Jan - 26-Jan)	30-Jan-20 (27-Jan - 02-Feb)	06-Feb-20 (04-Feb - 09-Feb)
Netherlands	27-Feb-20	06-Mar-20	12-Mar-20	13-Mar-20	-	24-Jan-20 (21-Jan - 27-Jan)	02-Feb-20 (30-Jan - 04-Feb)	10-Feb-20 (09-Feb - 12-Feb)
UK	30-Jan-20	05-Mar-20	16-Mar-20	17-Mar-20	Since late Feb^4^	28-Jan-20 (25-Jan - 31-Jan)	04-Feb-20 (02-Feb - 07-Feb)	12-Feb-20 (11-Feb - 14-Feb)
Belgium	02-Feb-20	11-Mar-20	13-Mar-20	17-Mar-20	Since late Feb^5^	29-Jan-20 (26-Jan - 01-Feb)	06-Feb-20 (04-Feb - 09-Feb)	15-Feb-20 (13-Feb - 17-Feb)
Germany	27-Jan-20	09-Mar-20	13-Mar-20	16-Mar-20	-	01-Feb-20 (30-Jan - 02-Feb)	09-Feb-20 (07-Feb - 10-Feb)	17-Feb-20 (16-Feb - 18-Feb)
New York	01-Mar-20	14-Mar-20	22-Mar-20	02-Feb-20	20-Feb-20^6^ (14-Feb - 26-Feb)	01-Feb-20 (29-Jan - 03-Feb)	08-Feb-20 (06-Feb - 10-Feb)	16-Feb-20 (14-Feb - 17-Feb)
Brazil	26-Feb-20	17-Mar-20	13-Mar-20	17-Mar-20	19-Feb-20^7^ (04-Feb - 28-Feb) 22-Feb-20^8^ (16-Feb - 27-Feb)	02-Feb-20 (29-Jan - 05-Feb)	10-Feb-20 (07-Feb - 13-Feb)	19-Feb-20 (17-Feb - 21-Feb)

*: source of data is specified on Supplementary data. 1: Lu et al. (2020); 2: Diez-Fuertes et al. (2020): clade G (B.1) in Europe; 3: Diez-Fuertes et al. (2020): clade S in Spain; 4: Pybus et al. (2020); 5: Dellicour et al. (2020); 6: Worobey et al. (2020); 7: Resende et al. (2020): clade B.1.1.BR; 8: Candido et al. (2020): clade 2; EGM: exponential growth model.

Genomic data shown that for some places the epidemic was mostly driven by few local predominant lineages (e.g., Spain, New York and Brazil), while for others it was probably seeded by a high number of importation events and no predominant lineage was identified so far (e.g., Belgium, Germany, Netherlands and the UK).^11^ ^,^ ^13^ ^,^ ^14^ ^,^ ^16^ ^,^ ^17^ ^,^ ^19^ ^,^ ^22^ ^,^ ^23^ To estimate the beginning of domestic spread in these locations, we made the TN10 and TN100 projections. The onset date of community transmission of SARS-CoV-2 was traced back to between late January and early February by the TN10 projections and to between early and mid-February by the TN100 projections (Fig. 2, Table). For Spain, NYC and Brazil, the TN10 projections point the origin of local viral transmissions 10-15 days earlier than the estimated T_MRCA_ of major transmission lineages detected (Table).^11^ ^,^ ^13^ ^,^ ^22^ ^,^ ^23^ For UK and Belgium, by its turn, the TN10 and TN100 projections recovered dates that were 15-20 days earlier than the estimated T_MRCA_ of local lineages detected (Table).^14^ ^,^ ^17^ The T_MRCA_ of local SARS-CoV-2 transmission lineages in France, Germany and Netherlands were not described so far. Thus, despite of accommodating for multiple seedings events, our projections of the onset date of community transmission of SARS-CoV-2 in most western countries provide timelines that were 10-20 days earlier than those estimated by molecular clock analyses (Table).

Overall, our results support a period of untracked (cryptic) community transmission of SARS-CoV-2 in Europe and the Americas from mid-January to late February 2020. According to our estimations, SARS-CoV-2 probably started to spread locally before community transmission was officially recognised and control measures for social distancing and air travel restrictions were implemented in all countries analysed and even before detection of the first imported cases in some locations (Fig. 2). The existence of sustained community transmission in Italy since the mid-January and in other western countries since late January or early February is consistent with the early establishment of the pandemic viral lineage B.1 in Italy and its rapid dissemination in Europe and the Americas.^11^ ^,^ ^22^ ^,^ ^23^ ^,^ ^24^ Our findings also agree with epidemiological data from syndromic surveillance of severe acute respiratory illness (SARI) that detected an excess of non-influenza SARI cases above the seasonal average in France, the USA and Brazil since late-February/early-March and further confirm SARS-CoV-2 positive samples among hospitalised SARI cases in Brazil since mid-February (16th-22nd February) (Available from: http://info.gripe.fiocruz.br).^9^ ^,^ ^41^ ^,^ ^42^

The TN1 projections and the molecular clock analyses provide quite convergent estimates of the probable onset date of community transmission of SARS-CoV-2 in those locations where the epidemic was mostly driven by one seeding event. For countries with evidence of few and multiple independent seeding events, however, our TN10 and TN100 projections push back the timeline of community spread 10-20 days earlier than molecular clock estimates, respectively. If epidemics in most western countries actually resulted from the concurrent dissemination of hundreds or thousands independent SARS-CoV-2 transmission lineages, then our projections will produce estimates systematically biased toward older dates. Alternatively, given the dissemination of identical (or very similar) SARS-CoV-2 strains across different western countries over a short-time interval, the T_MRCA_ of country-specific viral variants carrying one or a few synapomorphic mutations represent and upper bound of the time when SARS-CoV-2 became established in different locations. Interestingly, a recent study that estimate the establishment of local transmission of SARS-CoV-2 in the US using a metapopulation transmission model, concluded that the virus had been spreading in New York since the beginning of February, thus consistent with our TN1 or TN10 projections for this city and earlier than molecular clock estimates.^43^ These results suggest that the combined use of projections based on epidemiological and genomic data could provide a more accurate picture of the early SARS-CoV-2 epidemic history in different settings.

Our model has several limitations. First, the narrow confidence intervals of our estimates should be interpreted with caution because our method does not account for uncertainty and geographic variability in the mortality rate and lag-time between infection and death.^28^ ^,^ ^29^ As more clinical and epidemiological data becomes available, it will be possible to refine these estimates by using more accurate regional-specific parameters. Despite this, data from countries that implement wide-scale testing for SARS-CoV-2 since the beginning of the outbreak, including people who have mild or no symptoms, supports that the mortality rate will probably not exceed 1% of the total number of infected individuals.^44^ Second, our method is sensitive to underestimation of the true number of deaths from SARS-CoV-2 and does not take into account the time-delay between dead to report. Overestimation of the mortality rate, substantial underreporting of deaths, and/or significant time-delayed of deaths counts during the initial phase would lead an underestimation of the total number of infected individuals and, consequently, to more recent projections of the TN1, TN10 and TN100. Hence, the estimates presented here should be regarded as a conservative lower limit for the onset date of local spread.

In summary, our results suggest that community transmission of SARS-CoV-2 probably started in many western countries between mid-January to mid-February 2020, thus long before control measures to restrict air travels and promote social distancing were implemented. That quite long period of putative cryptic community transmission in Europe and the Americas draws attention to the great challenge of tracking the early global and local spread of SARS-CoV-2 and supports that control measures should be adopted at least as soon as first imported cases are detected in a new geographic region. This is especially important in the light of studies showing that SARS-CoV-2 very likely will enter in a regular circulation after the initial pandemic wave, causing recurrent outbreaks in the next years whose frequency and intensity are dependent upon virus’ biological features that are still not well understood, like the duration of immunity that SARS-CoV-2 can induce.^45^ Retrospective virological surveillance of people with SARI will be pivotal to trace the precise time of community transmission of SARS-CoV-2 in western countries, while active virological surveillance will be crucial for early detection of the virus re-emergence and rapid implementation of appropriate control measures.
