# Supplementary material for: Tracking the onset date of the community spread of SARS-CoV-2 in western countries
Source: Mem Inst Oswaldo Cruz. 2020 Sep 4;115:e200183. doi: 10.1590/0074-02760200183 (PMC7472723; doi:10.1590/0074-02760200183)
Supplement: Supplementary file 1 [file 1678-8060-mioc-115-e200183-s.pdf]

TABLE

Exponential growth equation and model fit

**Best-fit values**	**Italy**	**Spain**	**Brazil**	**France**	**UK**	**China**	**Netherlands**	**Belgium**	**Germany**	**New York**
Y0	2.09	3.20	5.04	2.62	1.36	4.09	2.55	2.22	1.11	7.03
k	0.30	0.31	0.27	0.24	0.30	0.25	0.261	0.27	0.29	0.31
EDT	2.31	2.21	2.59	2.92	2.28	2.82	2.65	2.58	2.42	2.25
**95% CI**										
Y0	1.61-2.57	1.52-4.89	3.50-6.58	1.89-3.34	0.77-1.95	2.54-5.64	1.66-3.45	1.31-3.13	0.82-1.39	4.62-9.44
k	0.29-0.31	0.28-0.35	0.24-0.30	0.22-0.25	0.28-0.33	0.22-0.27	0.24-0.28	0.25-0.29	0.27-0.30	0.29-0.33
EDT	2.21-2.41	2.00-2.46	2.32-2.93	2.74-3.13	2.09-2.50	2.57-3.13	2.45-2.88	2.37-2.82	2.30-2.54	2.09-2.43
**Goodness of Fit**										
R square	0.9977	0.9893	0.9896	0.9942	0.9919	0.9921	0.9932	0.9918	0.9968	0.994
**Wald-Wolfowitz runs test**										
P value	0.2867	0.4545	0.0714	0.2867	0.4545	0.0714	0.0545	0.1515	0.1369	0.2028
Deviation from Model	NS	NS	NS	NS	NS	NS	NS	NS	NS	NS

CI: confidence interval; EDT: epidemic doubling time; NS: not significant.

CC BY

Sources of the information about epidemiologic and control measures of COVID19

**1st recorded case**
https://www.who.int/docs/default-source/coronaviruse/situation-reports/20200121-sitrep-1-2019-ncov.pdf?sfvrsn=20a99c10_4<br>https://edition.cnn.com/2020/03/02/us/new-york-coronavirus-first-case/index.html<br>https://www.corriere.it/cronache/20_gennaio_30/coronavirus-italia-corona-9d6dc436-4343-11ea-bdc8-faf1f56f19b7.shtml<br>https://globalnews.ca/news/6485690/coronavirus-italy-first-two-cases/<br>https://elpais.com/sociedad/2020/01/31/actualidad/1580509404_469734.html<br>https://www.redaccionmedica.com/secciones/sanidad-hoy/coronavirus-infectados-espana-y-evolucion-covid19-desde-origen-4148<br>https://www.lefigaro.fr/sciences/coronavirus-trois-premiers-cas-confirmes-en-france-20200124<br>https://www.journalofinfection.com/article/S0163-4453(20)30102-X/fulltext<br>https://www.info-coronavirus.be/en/news/one-repatriated-belgian-has-tested-positive-for-the-novel-coronavirus/<br>https://berlinspectator.com/2020/03/26/chronology-germany-and-the-coronavirus-1/<br>https://www.stmgp.bayern.de/presse/bestaetigter-coronavirus-fall-in-bayern-infektionsschutzmassnahmen-laufen/<br>https://www.channelnewsasia.com/news/world/first-case-of-covid-19-confirmed-in-the-netherlands-12478958<br>https://www.rivm.nl/node/152811<br>https://espanol.medscape.com/verarticulo/5905076<br>https://g1.globo.com/sp/sao-paulo/noticia/2020/02/25/ministerio-da-saude-investiga-possivel-paciente-com-coronavirus-em-sp-caso-foipara-contraprova.ghtml
**1st recorded death**
https://www.aljazeera.com/news/2020/01/china-reports-death-mysterious-outbreak-wuhan-200111023325546.html<br>https://www.cnbc.com/2020/03/14/new-york-confirms-states-first-death-from-coronavirus.html<br>https://www.corriere.it/cronache/20_febbraio_21/coronavirus-muore-uomo-77-anni-monselice-dac529f6-54f9-11ea-9196-da7d305401b7.shtml<br>https://www.infobae.com/america/agencias/2020/03/21/hace-un-mes-que-parece-una-eternidad-italia-registro-su-primer-muerto-porcoronavirus/<br>https://www.abc.es/espana/comunidad-valenciana/abci-hombre-habia-contagiado-coronavirus-murio-13-febrero-valencia-202003032010_noticia.html<br>https://www.redaccionmedica.com/autonomias/valencia/primera-muerte-por-coronavirus-covid-19-en-espana-3106<br>https://www.bbc.com/mundo/noticias-51514703<br>https://globalnews.ca/news/6555510/france-coronavirus-death-europe/<br>https://www.bbc.com/news/uk-52082781<br>https://www.channelnewsasia.com/news/world/first-covid-19-death-reported-in-belgium-12526942<br>https://www.garda.com/crisis24/news-alerts/321691/belgium-government-confirms-first-covid-19-related-death-march-11-update-2<br>https://www.straitstimes.com/world/europe/germany-reports-first-death-cases-due-to-coronavirus<br>https://www.aa.com.tr/en/europe/coronavirus-germany-reports-first-2-deaths/1759875<br>https://www.garda.com/crisis24/news-alerts/320336/netherlands-first-covid-19-death-confirmed-march-6-update-3<br>https://www.rivm.nl/nieuws/patient-met-nieuw-coronavirus-overleden<br>https://www.clarin.com/mundo/brasil-registro-primera-muerte-coronavirus_0_AhLtmut2.html &<br>https://www.aa.com.tr/es/mundo/brasil-confirma-el-primer-muerto-por-covid-19/1769401
**Internal movement**
https://science.sciencemag.org/content/early/2020/03/30/science.abb6105<br>https://www.thedailybeast.com/new-york-gov-andrew-cuomo-implements-stay-at-home-order-amid-coronavirus-covid-19-pandemic<br>https://www.theguardian.com/world/2020/feb/23/coronavirus-northern-italian-towns-close-schools-and-businesses<br>https://elpais.com/espana/2020-03-14/el-gobierno-prohibe-todos-los-viajes-que-no-sean-de-fuerza-mayor.html<br>https://www.thelocal.fr/20200316/lockdown-what-exactly-do-frances-new-coronavirus-rules-mean<br>https://www.pinsentmasons.com/out-law/analysis/coronavirus-uk-lockdown<br>https://www.euractiv.com/section/coronavirus/news/belgium-enters-lockdown-over-coronavirus-crisis-until-5-april/<br>https://www.france24.com/en/20200317-merkel-announces-strict-measures-and-tells-germans-to-stay-home-in-virus-fight<br>https://www.dw.com/en/coronavirus-germany-brings-in-unprecedented-restrictions-on-public-life/a-52801471<br>https://www.politico.eu/article/dutch-pm-mark-rutte-we-wont-impose-national-lockdown-coronavirus-covid19/<br>https://governo-sp.jusbrasil.com.br/legislacao/821536913/decreto-64862-13-marco-2020-sao-paulo-sp<br>http://www.aeerj.net.br/file/16-03-2020-corona.pdf
